# Supplementary material for: Dynamic transcriptomic profiles of zebrafish gills in response to zinc supplementation
Source: BMC Genomics. 2010 Oct 11;11:553. doi: 10.1186/1471-2164-11-553 (PMC3091702; doi:10.1186/1471-2164-11-553)
Supplement: Additional file 2 — Interactive Direct Interaction Network representing the molecular interactions between zinc, copper, iron, calcium and proteins encoded by transcripts changed by zinc supplementation. Mini web-site containing index.html and hyperlinked pages in subdirectory describing a Direct Interaction Network automatically generated based on curated interactions contained within the proprietary PathwayArchitect database. Ovals represent proteins and the circles symbolize metal ions. Objects are coloured by their abundance in zebrafish at the time-point they were significantly different from the control is a scale from -4 fold (dark green) to +4 fold (dark red). Where significant differences were found at more than one time-point, the colour overlay shows expression at the first instance. Dark blue squares denote 'binding', and light blue squares 'expression'; green squares stand for 'regulation', green diamonds for 'metabolism', and green circles for 'promoter binding'. Arrow heads indicate directionality of the interaction where annotated. All nodes and edges can be further interrogated by selecting the relative area of the image. [file 1471-2164-11-553-S2.zip › PathwayArchitect Zn xs DIN/142607.html]

# PROTEIN: CYP11A1

|  |  |
| --- | --- |
| Name | CYP11A1 |
| Type | PROTEIN |
| Description | cytochrome P450, family 11, subfamily A, polypeptide 1 |
| Note | This gene encodes a member of the cytochrome P450 superfamily of enzymes. The cytochrome P450 proteins are monooxygenases which catalyze many reactions involved in drug metabolism and synthesis of cholesterol, steroids and other lipids. This protein localizes to the mitochondrial inner membrane and catalyzes the conversion of cholesterol to pregnenolone, the first and rate-limiting step in the synthesis of the steroid hormones. |
| Alias | Cyp11a |
|  | cholesterol side-chain cleavage cytochrome P450 |
|  | Cholesterol side-chain cleavage enzyme |
|  | cytochrome P450 11a cholesterol side |
|  | cytochrome P450 subfamily 11A |
|  | CYP11A |
|  | P450SCC |
|  | cholesterol desmolase |
|  | cytochrome P450, subfamily XIA (cholesterol side chain cleavage) |
|  | cytochrome P450 side-chain cleavage enzyme |
|  | steroid 20-22-lyase |
|  | cholesterol monooxygenase (side-chain cleaving) |
|  | cytochrome P450 11a cholesterol side chain cleavage |
|  | cholesterol side chain cleavage |
|  | Cholesterol desmolase |
|  | Cyp11a-1 |
|  | cytochrome P450, subfamily XIA |
|  | CYPXIA1 |
|  | cytochrome P450C11A1 |
|  | cholesterol 20-22 desmolase |
|  | cholesterol side-chain cleavage enzyme |
|  | CYP11A1 |
|  | Cypxia1 |
|  | P450(scc) |
|  | D9Ertd411e |
|  | Cyp11a1 |
|  | cytochrome P450, subfamily 11A |
|  | Scc |
|  | P450scc |
|  | cytochrome P450, 11a, cholesterol side-chain cleavage |


---

|  |  |
| --- | --- |
| GO Component | mitochondrion |
|  | membrane |


---

|  |  |
| --- | --- |
| GO ID | GO:0016020 |
|  | GO:0020037 |
|  | GO:0008203 |
|  | GO:0005506 |
|  | GO:0005489 |
|  | GO:0006118 |
|  | GO:0019825 |
|  | GO:0006702 |
|  | GO:0008202 |
|  | GO:0046872 |
|  | GO:0006700 |
|  | GO:0008386 |
|  | GO:0004497 |
|  | GO:0005739 |
|  | GO:0016491 |
|  | GO:0006629 |


---

|  |  |
| --- | --- |
| MIM | MIM:118485 |


---

|  |  |
| --- | --- |
| Connectivity | 447 |


---

|  |  |
| --- | --- |
| Entrez ID | 29680 |
|  | 1583 |
|  | 13070 |


---

|  |  |
| --- | --- |
| Agilent ID | A\_51\_P309733 |
|  | A\_51\_P309727 |
|  | A\_53\_P136981 |
|  | A\_43\_P11896 |
|  | A\_23\_P129169 |
|  | A\_42\_P636350 |
|  | A\_51\_P309731 |
|  | A\_23\_P129167 |
|  | A\_53\_P165635 |
|  | A\_14\_P100556 |


---

|  |  |
| --- | --- |
| Cellular Localization | Mitochondrion |
|  | Membrane |
|  | Cell |
|  | Cytoplasm |
|  | Organelle |


---

|  |  |
| --- | --- |
| DbXref | KEGG pathway##00140##C21-Steroid hormone metabolism##http://www.genome.jp/dbget-bin/show\_pathway?mmu00140+13070 |
|  | KEGG pathway##00140##C21-Steroid hormone metabolism##http://www.genome.jp/dbget-bin/show\_pathway?rno00140+29680 |
|  | KEGG pathway##00140##C21-Steroid hormone metabolism##http://www.genome.jp/dbget-bin/show\_pathway?hsa00140+1583 |


---

|  |  |
| --- | --- |
| Pathway | Zn xs inventory |
|  | Zn xs DIN |


---

|  |  |
| --- | --- |
| GO Process | cholesterol metabolism |
|  | electron transport |
|  | androgen biosynthesis |
|  | C21-steroid hormone biosynthesis |
|  | lipid metabolism |
|  | steroid metabolism |


---

|  |  |
| --- | --- |
| UniGene | Mm.302865 |
|  | Rn.1401 |
|  | Hs.303980 |


---

|  |  |
| --- | --- |
| Affymetrix Probeset ID | 1368468\_at |
|  | 1439947\_at |
|  | 1448804\_at |
|  | 204309\_at |
|  | 37195\_at |
|  | 96228\_at |
|  | AA389018\_s\_at |
|  | g4503188\_3p\_at |
|  | J05156\_s\_at |
|  | M14565\_at |
|  | 1458194\_at |
|  | 1457604\_x\_at |
|  | 1384456\_at |
|  | 43526\_at |
|  | 67230\_at |
|  | RC\_AA039461\_at |
|  | RC\_T53590\_s\_at |
|  | TC32455\_at |
|  | TC33376\_at |
|  | rc\_AI029527\_at |


---

|  |  |
| --- | --- |
| EC Number | EC 1.14.15.6 |


---

|  |  |
| --- | --- |
| GO Function | cholesterol monooxygenase (side-chain-cleaving) activity |
|  | heme binding |
|  | oxygen binding |
|  | oxidoreductase activity |
|  | monooxygenase activity |
|  | iron ion binding |
|  | electron transporter activity |
|  | metal ion binding |


---

|  |  |
| --- | --- |
| Nucleotide | J05511 |
|  | NM\_019779 |
|  | BC068264 |
|  | AF195119 |
|  | J05156 |
|  | M63133 |
|  | AK056794 |
|  | AK042878 |
|  | BC089100 |
|  | M22615 |
|  | BC032329 |
|  | M28253 |
|  | X05367 |
|  | AK135608 |
|  | AK137397 |
|  | AK163559 |
|  | NM\_017286 |
|  | M14565 |
|  | X14257 |
|  | NM\_000781 |


---

|  |  |
| --- | --- |
| Protein | AAN86648 |
|  | AAH32329 |
|  | NP\_062753 |
|  | AAA52162 |
|  | AAA40989 |
|  | Q9QZ82 |
|  | AAF03897 |
|  | P05108 |
|  | NP\_058982 |
|  | AAA36404 |
|  | P14137 |
|  | AAA40958 |
|  | CAA28965 |
|  | AAH89100 |
|  | NP\_000772 |
|  | AAA62267 |
|  | CAA32471 |
|  | AAH68264 |


---

|  |  |
| --- | --- |
| Organism | Mammal |


---

|  |  |
| --- | --- |
| Location | chromosome 9, 9 31.0 cM, 9 B (Mus musculus) |
|  | 9 31.0 cM (Mus musculus) |
|  | chromosome 8, 8q24 (Rattus norvegicus) |
|  | chromosome 15, 15q23-q24 (Homo sapiens) |


---

|  |  |
| --- | --- |
